# Two Experimental Protocols for Accurate Measurement of Gas Component Uptake and Production Rates in Bioconversion Processes

**DOI:** 10.1038/s41598-019-42469-3

**Published:** 2019-04-11

**Authors:** Kyle A. Stone, Q. Peter He, Jin Wang

**Affiliations:** Department of Chemical Engineering, Auburn University, Auburn, AL USA 36849

## Abstract

Bioconversion processes offer many economic, environmental, and societal advantages for production of fuels and chemicals. Successful commercialization of any biotechnology usually requires accurate characterization of cell growth dynamics, substrate conversion and production excretion rates. Despite recent advancements in analytical equipment, obtaining accurate measurement of gas component uptake or production rates remains challenging due to their high sensitivity to system pressure or volume changes. Specifically, the consumption and production of various gases will result in changes in system pressure (for batch operations) or off-gas flow rate (for continuous operations). These changes would cause significant errors in the estimated gas component uptake and production rates if they were not accounted for. In this work, we propose two easy-to-implement experimental protocols and associated calculation procedures to obtain accurate measurements of gas component consumption and production rates; one is for batch operation and one is for continuous operation. For depressurized (*i*.*e*., system pressure below 1 atm) batch cultures, nitrogen (or other inert gases) is used to repressurize the system to 1 atm before taking sample; while for continuous cultures, He (or other inert gases) is used as an internal tracer to accurately measure off-gas flow rate. The effectiveness and accuracy of the two protocols and associated calculation procedures are demonstrated using several case studies with both abiotic and biotic systems.

## Introduction

Compared to traditional chemical and thermochemical manufacturing processes, bioconversion processes offer several distinct advantages. These advantages include the ability to operate at ambient temperatures and pressures, high specificity and selectivity, etc. Therefore, bioconversion offers a viable or even preferred option to traditional chemical production. Sometimes large-scale facility is not an economically or technically feasible option due to the lack of high-volume feedstocks and/or lack of viable transportation infrastructure^1^. Bioconversion is particularly attractive for such ceases. In addition, with recent advancement in metabolic engineering and industrial process design, exploiting the diversity and strengths of biological processes could offer considerable economic, environmental, and societal advantages.

Successful commercialization of any potential biotechnology requires advanced knowledge and understanding of the fundamental biological conversion steps within the microorganism. To acquire these knowledge, it requires accurate characterization of cell growth dynamics, substrate conversion and production excretion rates. Recent advancement in analytical equipment such as NIR spectroscopy, Raman spectroscopy, and NMR, has enabled fast and accurate measurement of many excreted metabolites in the liquid broth. In addition, tracking carbon flow within the cell through ^13^C labeling also become a commonly utilized tool. However, accurate measurement of gas component uptake or production rates remains challenging due to their high sensitivity to system pressure or volume changes. This problem is widespread as most bioconversion processes involve gas components, such as aerobic growth, syngas fermentation, and photosynthesis, etc. If accurate measurements of gas component consumption and production rates can be obtained, it will have broad applications for characterizing cell growth dynamics, substrate conversion and production excretion rates. More importantly, it will enable better and quantitative understanding of the biocatalyst’s cellular metabolism through genome-scale metabolic network models (GEMs) as the cross membrane metabolic fluxes are commonly used constrains for GEMs development and refinement^2^.

The challenges with measuring gas component consumption and production rates are not caused by the precision of analytical equipment (*e*.*g*., gas chromatography). Instead, they are rooted in the fact that the consumption and/or production of gases alter the system headspace pressure (for batch processes) or gas phase flow rate (for continuous processes). Such pressure or flow rate changes have not been explicitly or accurately accounted for in the available protocols for gas component measurements. For example, research on methanotrophs offers an excellent example on the importance of accurate gas measurement, as these microbes CH_4_ and O_2_ to produce biomass and other organic compounds. However, among recent publications on methanotrophs, there was no mentioning on how system pressure change would affect gas phase measurements for batch experiments^3^, or how off-gas flow rate change would affect gas phase measurements for continuous experiments^4,5^. There were only simple descriptions on what equipment was used for gas composition measurement. Among references we examined, only one paper briefly mentioned that “the outlet flow rate during the (continuous) experiment was determined by completing a N_2_ balance^6^”. However, there was no details provided, nor the accuracy of the measurement examined or validated.

**Figure 1:**
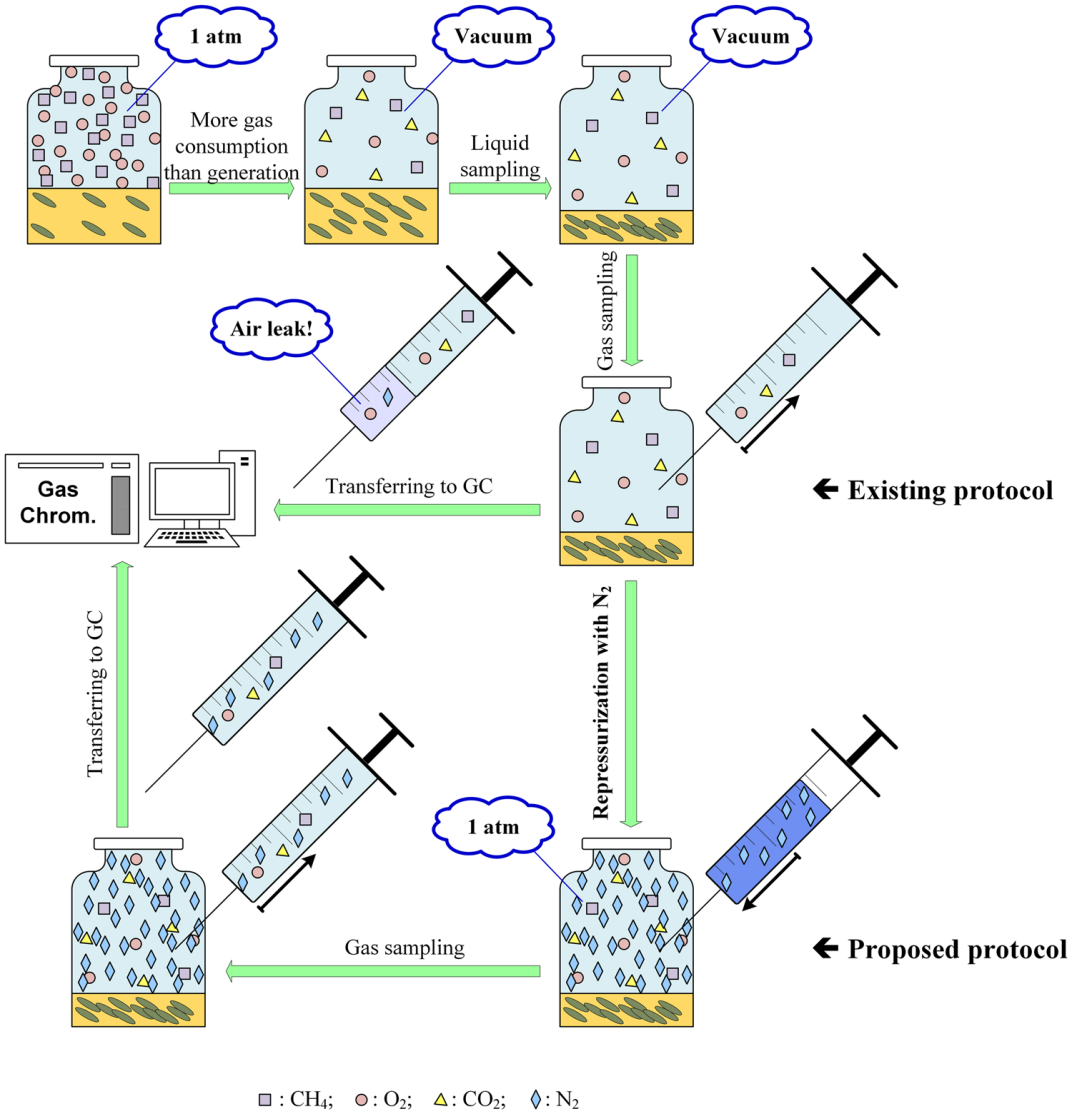
Existing protocol of measuring gas composition for depressurized systems can cause significant error, especially for O_2_ consumption rate, because of the air leakage into the sampling syringe due to vacuum pressure of the system. The proposed protocol of repressurization with an inert gas such as N_2_ or He will overcome this issue. (Illustrative system of methane oxidation by methanotrophs).

Here we first explain where the major measurement error usually occurs for gas phase components in batch and continuous operations. For batch experiments that are conducted in closed-systems with constant volume such as vials, the system pressure often experiences significant reduction. This can be caused by the overall gas consumption greater than gas production, as well as gas and/or liquid sampling. As shown in Fig. 1, when a headspace gas sample is taken from such a vacuum system with a gas-tight syringe, ambient air enters the syringe after it is withdrawn from the system due to the vacuum pressure in the system. As illustrated in this work, such change could cause significant errors in the measured gas compositions. On the other hand, pressurized system (*i*.*e*., system pressure above 1 atm) can be handled relatively easily. For pressured systems, the error caused by gas sample exiting the syringe can be corrected by measuring the system pressure (denoted by *P*) and scale the measured composition back up by $\frac{P}{1\,atm}$. Therefore, in this work, we focus on addressing the case of depressurized systems with vacuum pressure.

For continuous chemostat operations, the system pressure is constant. However, due to the often imbalance between gaseous substrate consumption and gaseous product excretion, the off-gas flow rate can be significantly different from the feeding gas flow rate. In order to estimate various gas consumption and production rates, it is critical to accurately measure off-gas flow rate. Although mass flow controllers are commonly used to measure and control the inlet gas flow rate accurately, they are not as effective for off-gas flow rate. This is because the mass flow meters have to be calibrated with known gas composition to obtain accurate measurements of gas mixtures, but off-gas composition often changes overtime and is usually unknown before experiment.

In this work, we present two easy-to-implement experimental protocols along with the calculation procedures to measure gas phase component concentrations for depressurized batch experiments and off-gas flow rate for continuous experiments. For this study, an aerobic methanotroph (*Methylomicrobium buryatense* 5GB1) serves as the model culture as it utilizes gaseous methane as the sole carbon and energy sources, consumes O_2_ and produces CO_2_ as a major product^4,7,8^.

## Methods

### Repressurization protocol for measuring gas component concentrations in depressurized batch operations

For depressurized batch cultures, to prevent ambient air from entering the sampling syringe after withdrawn from the system, we first repressurize the system to atmosphere pressure using an inert gas that has negligible effect on cell growth and gas measurement (such as N_2_ or He). Next, a gas sample is taken and measured using GC (Agilent 7890B customized with FID, TCD, Unibeads IS 60/80 mesh and MolSieve 5A 60/80 SST columns). The procedure is illustrated in the lower part of Fig. 1. Although it has been reported that methanotroph could fix N_2_, the brief contact time for the batch repressurization protocol is too short for potential N_2_ fixation to cause any noticeable error in the measurement of different gas components. Therefore, N_2_ can be used as an inert gas for the repressurization protocol. In this work, we tested both N_2_ and He as inert gas to repressurize the system, and the obtained results were consistent with each other. The results presented in this work for batch repressurization protocol were obtained using N_2_, which is the carrier gas of our GC system.

It is worth noting that this repressurization increases the system pressure without changing the molar concentrations of the interested gas components (such as CH_4_, and O_2_). In other words, the GC measured molar concentration (mmol/L in this work) following the repressurization protocol is the true molar concentration in the originally depressurized system. This is because for most gases such as O_2_ and CH_4_, they all have very high Henry’s constants, which means that their solubilities in culture broth (assumed similar to their solubilities in water) are very low and the solubility changes due to the small pressure change during repressurization would be negligible. However, this is not the case for CO_2_ in buffered solutions, and we track CO_2_ in a buffered solution through measuring the total inorganic carbon of the liquid phase as described later.

### The helium tracer protocol for measuring off-gas flow rate in continuous and semi-continuous operations

For continuous operation of bioreactors, we use He as a tracer to determine the off-gas flow rate. Specifically, He gas is included in the feed gas at a fixed volumetric fraction (10 vol% in this work) to serve as an internal standard for the bioconversion of methane. Concentration of He in the gas stream can be accurately measured using GC as N_2_ is the carrier gas in our GC system. Helium is selected as the internal standard for the following reasons: (1) it is an inert gas with no toxic effect on cell growth; (2) it has negligible solubility in water at 1 atm and 37 °C^9^. Again, other inert gases could be utilized as the tracer provided that they do not interfere with the system and their concentrations can be measured accurately. However, we do not recommend using N_2_ as the internal tracer to estimate off-gas flow rate. Because methanotrophs can fix N_2_ as their nitrogen source, the amount of N_2_ in the off-gas may be different from that in the feeding gas. As He is neither consumed nor dissolved in the liquid, the mole balance of He enables us to determine the off-gas flow rate. Since the molar flow rate of He in the feed equals to the molar flow rate of He in the off-gas, the off-gas flow rate can be calculated through the measured molar concentration of He, as shown below:

1 $${Q}_{Total,\,{\rm{out}}}^{g}=\frac{{y}_{He,{\rm{in}}}}{{y}_{He,{\rm{out}}}}{Q}_{Total,{\rm{in}}}^{g}=\frac{{y}_{He,{\rm{in}}}P}{{C}_{He,{\rm{out}}}^{g}RT}{Q}_{Total,{\rm{in}}}^{g}$$

where ${Q}_{Total,\,{\rm{in}}}^{g}$ and ${Q}_{Total,\,{\rm{out}}}^{g}$ denote the total volumetric flow rates of feed gas and off-gas, respectively; ${y}_{He,{\rm{in}}}$ and ${y}_{He,{\rm{out}}}$ denote the molar fractions of He in the feed gas and off-gas, respectively, which are the same as the volumetric fractions when the process is at constant pressure such as 1 atm in this work. ${y}_{He,{\rm{in}}}$ is obtained based on the flow rates of all gas components in the feed gas. ${C}_{He,{\rm{out}}}^{g}$ is the molar concentration of He in off-gas measured by GC (mmol/L); *P* is the system pressure, which is 1 atm in this work; *T* is the room temperature that the GC is calibrated, which is 294.15 K in this work; *R* is the universal gas constant, which is $8.206\times {10}^{-5}\frac{L\,atm}{mmol\,K}$. Because all the quantities on the right-hand side of Eq. (1) are known or measured, ${Q}_{f}^{g}$ can be directly calculated from Eq. (1). It is worth noting that Eq. (1) is also valid for total molar flow rate: ${F}_{Total,\,{\rm{out}}}^{g}=\frac{{y}_{He,{\rm{in}}}}{{y}_{He,{\rm{out}}}}{F}_{Total,{\rm{in}}}^{g}=\frac{{y}_{He,{\rm{in}}}P}{{C}_{He,{\rm{out}}}^{g}RT}{F}_{Total,{\rm{in}}}^{g}$.

### Estimating and measuring dissolved gases in liquid phase

#### Estimating dissolved gas concentration

For certain gas components, the amount of the dissolved gases in liquid can be accurately computed from gas phase concentrations based on Henry’s law. This is the case for gas components whose dissolved forms in liquid phase are predominantly the same molecules as in gas phase, and the approximate equilibrium of the dissolving process is reached quickly (*i*.*e*., within a few minutes). Many gases fall into this case, such as CH_4_, O_2_, N_2_, CO. It is also the case for CO_2_ in unbuffered neutral aqueous solution (pH at 7), as the dissolved CO_2_ remains predominantly as CO_2_ molecules with less than 0.2% converted to carbonic acid^10^.

There are many variants of Henry’s law constants. In this work, we use the dimensionless Henry solubility ${H}^{cc}$, a.k.a. water–air partitioning coefficient, which is defined as the ratio between the liquid-phase concentration ${C}^{l}\,$of a species and its gas-phase concentration *C*^*g*^, *i*.*e*., ${H}^{cc}={C}^{l}/{C}^{g}$. Once the gas-phase concentration is determined by GC, the liquid-phase concentration is calculated by ${C}^{l}={C}^{g}{H}^{cc}$. ${H}^{cc}$ of the gases used in this study are listed in Supplementary Information S1^11^.

#### Measuring dissolved CO_2_ concentration

Henry’s law does not apply for CO_2_ in alkaline or buffered medium for biotic systems, where the disassociation of molecular CO_2_ to bicarbonate and carbonate salts become much more prevalent^12^. When the pH of the solution is not tightly controlled, it is not possible to obtain accurate estimation of the dissolved CO_2_ in liquid phase through Henry’s law, as the equilibrium among H_2_CO_3_, HCO_3_^−^ and CO_3_^2−^ are very sensitive to the solution pH. To address this challenge, we first remove cell mass through centrifugation, then measure the total inorganic carbon (TIC) of the liquid phase via a Shimadzu TOC-V_CSN_ analyzer. Because the TIC includes all forms of inorganic carbon, it is not affected by the equilibrium among H_2_CO_3_, HCO_3_^−^, CO_3_^2−^, and dissolved molecular CO_2_. Therefore, the change in TIC between two time points reflects the net amount of CO_2_ dissolved in or vaporized from the liquid phase during that period of time, regardless of the balance shift among different dissolved forms. It is worth noting that because the background TIC, *i*.*e*., TIC in the feeding medium, is measured and subtracted from following samples, it will not introduce error into the calculation of dissolved CO_2_.

## Experiments

### Abiotic batch experiments comparing existing and proposed protocols

Two abiotic experiments were conducted to compare the existing protocol with the proposed protocol.

#### Experiment 1

This experiment was conducted following the existing protocol, *i*.*e*., the headspace is sampled using a gas tight syringe without repressurization. First, serum vials (250 mL) were filled with 150 mL of distilled water and flushed with feeding gas mixture (10% CH_4_, 25% CO_2_, 25% O_2_ and 40% N_2_) at 200 smL/min for 10 minutes (smL denotes standard milliliter with reference temperature of 20 °C (68 °F) and pressure of 1 atm). All percentages are volumetric percentages (vol%) in this work unless otherwise specified. After gas feeding, the head space is sampled using a gastight syringe (Hamilton) and measured with GC (Agilent Technologies 7890B GC system with FID/TCD). This composition is denoted as initial gas composition. Next, 40 mL of water is carefully removed from each vial using a syringe, to simulate the vacuum caused by gas consumption and liquid sampling. Finally, the head space is sampled again without repressurization using a gastight syringe and measured again using the GC. This composition is terms final composition. Due to the vacuum pressure in the vial, ambient air would enter into the syringe when the gas sample is transferred from the vial to the GC. Duplicate experiments were carried out, and single gas sample was taken for the initial and final GC gas composition measurements. The exact volume of water added/removed was determined through weighting the vials before and after the adding/removing of water to ensure its accuracy.

#### Experiment 2

This experiment was conducted following the proposed protocol, *i*.*e*., the gas sample is taken from the headspace after it is repressurized to 1 atm with N_2_. First, 250 mL serum vials were filled with 150 mL of distilled water and flushed with feeding gas mixture (7.5% CH_4_, 17% O_2_, 20% CO_2_, and 55% N_2_,) at 200 smL/min for 5 minutes. Then initial gas composition was measured through GC. Next about 100 mL of water was removed carefully, and the vials were repressurized using N_2_ to 1 atm before gas samples were taken and measured with GC. Because of the repressurization, there would not be any leakage into or from syringe when the gas sample is transferred from the vial to the GC. Duplicate experiments were carried out, and single gas sample was taken for the initial and final GC gas composition measurements.

### Evaluating measurement accuracy through mole balance for abiotic systems

For the above experiments with abiotic systems, the accuracy of the analytical protocols can be evaluated through a general mole balance by comparing the total final amount with the total initial amount for each gas component. Below we use CH_4_ as an example to illustrate how the mole balance is carried out. The mole balances for O_2_ and CO_2_ follow the same procedure.

For the initial state, the total amount (in mmol) of CH_4_ (${N}_{C{H}_{4},0}$) consists of the CH_4_ in gas phase (${N}_{C{H}_{4},0}^{g}$) and dissolved CH_4_ in liquid phase (${N}_{C{H}_{4},0}^{l}$). The gas phase amount is computed through GC measured initial molar concentration (${C}_{C{H}_{4},0}^{g}$) multiplied with the initial gas phase volume (${V}_{0}^{g}$), and the dissolved amount is determined through initial liquid molar concentration (${C}_{C{H}_{4},0}^{l}={C}_{C{H}_{4},0}^{g}{H}_{C{H}_{4}}^{cc}$, based on Henry’s law) multiplied with the initial liquid volume (${V}_{0}^{l}$):

2 $${N}_{C{H}_{4},0}={N}_{C{H}_{4},0}^{g}+{N}_{C{H}_{4},0}^{l}={C}_{C{H}_{4},0}^{g}{V}_{0}^{g}+{C}_{C{H}_{4},0}^{l}{V}_{0}^{l}={C}_{C{H}_{4},0}^{g}{V}_{0}^{g}+({C}_{C{H}_{4},0}^{g}{H}_{C{H}_{4}}^{cc}){V}_{0}^{l}$$

For the final state after the water removal, the total amount of CH_4_ ($${N}_{C{H}_{4},f}$$) consists of three parts: CH_4_ in the gas phase ($${N}_{C{H}_{4}\,,f}^{g}$$), in the liquid phase ($${N}_{C{H}_{4}\,,f}^{l}),\,\,$$and in the removed liquid ($${N}_{C{H}_{4}}^{r})$$. $${N}_{C{H}_{4}\,,f}^{g}$$ and $${N}_{C{H}_{4}\,,f}^{l}$$ are calculated similarly to those of the initial state but with the GC measured final gas composition. $${N}_{C{H}_{4}}^{r}$$ is determined using the initial composition as the liquid withdrawing process was fast, so it is assumed that the liquid removed was in equilibrium with the initial gas phase.

3 $$\begin{array}{c}{N}_{C{H}_{4},f}={N}_{C{H}_{4},f}^{g}+{N}_{C{H}_{4},f}^{l}+{N}_{C{H}_{4}}^{r}={C}_{C{H}_{4},f}^{g}{V}_{f}^{g}+{C}_{C{H}_{4},f}^{l}{V}_{f}^{l}+{C}_{C{H}_{4}}^{r}{V}^{r}\\ \,\,\,\,\,=\,{V}_{C{H}_{4},f}^{g}{V}_{f}^{g}+({V}_{C{H}_{4},f}^{g}{H}_{C{H}_{4}}^{cc}){V}_{f}^{l}+({V}_{C{H}_{4},0}^{g}{H}_{C{H}_{4}}^{cc}){V}^{r}\end{array}$$

where ${V}_{f}^{g}$, ${V}_{f}^{l}\,$and ${V}^{r}$ are the volumes of the final gas phase, final liquid phase, and removed liquid respectively.

Based on mole balance, ${N}_{C{H}_{4},0}={N}_{C{H}_{4},f}$. Therefore, the percentage error between initial and final total amounts as defined below can be used to assess the accuracy of the measurement procedure.

4 $$Err \% =\frac{{N}_{C{H}_{4},f}-{N}_{C{H}_{4},0}}{{N}_{C{H}_{4},0}}\times 100 \% $$

The accuracies for O_2_ and CO_2_ measurements are assessed following the same procedure. An example showing the detailed calculation procedure is provided in Supplementary Information S2.

### Biotic batch experiment with *Methylomicrobium buryatense* 5GB1

This experiment was designed to test the repressurization protocol on a biotic system of methane conversion with *M*. *buryatense* 5GB1 under batch operation. The strain was provided by Dr. Mary Lidstrom (University of Washington) and grown in modified nitrate mineral salts (NMS2) medium as described by Puri *et al*.^4^. First, 250 mL serum vials were filled with 50 mL of medium, tapped with a rubber septum and crimped with an aluminum cap. Then a gas mixture of CH_4_, O_2_ and N_2_ was fed continuously at 200 smL/min for 10 minutes. Totally 3 different gas compositions were tested with fixed CH_4_% (20%) but varying O_2_% (20%, 40%, 60%), which correspond to the O_2_:CH_4_ ratio of 1:1, 2:1 and 3:1, respectively. N_2_ was used to make up the rest of the composition, *i*.*e*., 60%, 40% and 20%, respectively. After feeding, the initial gas sample is taken from the vials to measure the gas phase composition via GC. Next, the vials were inoculated with pre-cultured *M*. *buryatense* 5GB1 cells that were grown till mid-exponential growth phase, to obtain an initial cell concentration of 0.05–0.06 gDCW/L. After inoculation, the first liquid sample is taken to obtain the initial optical density (OD) via a UV/Vis spectrophotometer (Beckman Coulter DU® 730); then after removing cells in the liquid sample through centrifugation, the baseline total carbon (TC) and total inorganic carbon (TIC) were measured via a TOC analyzer (Shimadzu TOC/VCSN). Additional samples were taken every 4 hours during the day of experimentation and 8 hours overnight. Because of the overall higher gas (*i*.*e*., CH_4_ and CO_2_) consumption rate than the gas (*i*.*e*., CO_2_) production rate, the vial were in vacuum pressure state. The proposed repressurization protocol with N_2_ was followed before gas sampling to prevent air leakage during sample transfer from vial to GC. All experiments were conducted in duplicates, and one liquid sample was taken for each time point, with the gas sample taken for the final sampling point.

### Biotic continuous experiment with *M*. *buryatense* 5GB1

This experiment was designed to test the proposed He tracer protocol on a biotic system of methane conversion with *M*. *buryatense* 5GB1 under continuous operation. All experiments were conducted in an Eppendorf Bioflo 115 with a working volume of 1.5 L. A methane-limited condition and an oxygen-limited condition were tested. The feeding gas flow rate was controlled by a mass flow controller at 300 smL/min and delivered through a microsparger. The feeding gas included 10% He gas as the tracer. Agitation was fixed at 500 rpm; temperature was controlled at 30 °C and pH was maintained at 9 via addition of 4 M NaOH. The liquid medium continuously fed into the reactor was NMS2 medium without buffer, but with double amount of nitrate and trace element to avoid nutrient limitation^3^. Antifoam (Struktol J 660 R) was continuously added to the reactor through a syringe pump (New Era, Farmingdale, NY) to control the foam generated. After inoculation, the bioreactor was operated under batch mode with continuous gas feeding to accumulate biomass, and switched to continuous operation once cell density is above 1 gDCW/L. Once a steady state was obtained, it was maintained for 3 to 4 days, then feeding gas composition or dilution rate was adjusted to reach a different steady state.

### Evaluating measurement accuracy through total carbon balance for biotic systems

Evaluating measurement accuracy is more complicated for biotic systems, as CH_4_ is converted to different products by the cells. These products include biomass, CO_2_ and excreted organic compounds such as various organic acids and extracellular polymeric substances (EPS). However, mole balance for carbon still holds, which offers the basis to examine the accuracy of the obtained measurements. Specifically, the amount of carbon consumed (${N}_{C,Consumed}$, in mmol) through CH_4_ assimilation should equal to the amount of carbon contained in different products produced (${N}_{C,Produced}$) during the same period.

For batch experiments, the amount of carbon consumed between two sampling points can be calculated through the difference between the initial and final gas phase compositions between the two sampling points, as shown below

5 $${N}_{C,Consumed}=-\,{\rm{\Delta }}{N}_{C{H}_{4}}^{g}={C}_{C{H}_{4},0}^{g}{V}_{0}^{g}-{C}_{C{H}_{4},f}^{g}{V}_{f}^{g}$$

where ${V}_{0}^{g}$ and ${V}_{f}^{g}$ are the initial and final gas phase volumes (in mL) respectively. Eq. (5) ignores the change of CH_4_ in the liquid phase because of the small Henry solubility (${H}_{i}^{cc}$) for CH_4_. The amount of produced carbon between two sampling points consists of three parts as shown in Eq. (6), corresponding to carbon contained in the produced biomass, CO_2_ and excreted organic compounds. In this work, carbon contained in biomass (${N}_{BC}$) was determined by the biomass equation used in a genome-scale metabolic model of *M*. *buryatense* 5GB1 with a conversion coefficient of 39.3 mmol carbon per gDCW^5^; The amount of CO_2_ produced were captured by the summation of gas phase CO_2_ amount changes (${\rm{\Delta }}{N}_{C{O}_{2}}^{g}$) and the TIC amount changes in the liquid phase (${\rm{\Delta }}{N}_{TIC}$) between two sampling points; The carbon contained in various excreted organic compounds (OC) were tracked through total organic compound (TOC) measurement which is the difference between total carbon and total inorganic carbon, TOC = TC − TIC. Therefore, the total amount of produced carbon between two sampling points is calculated as the following.

6 $$\begin{array}{rcl}{N}_{C,Produced} & = & {\rm{\Delta }}{N}_{BC}+{\rm{\Delta }}{N}_{C{O}_{2}}^{g}+{\rm{\Delta }}{N}_{TIC}+{\rm{\Delta }}{N}_{TOC}={\rm{\Delta }}{N}_{BC}+{\rm{\Delta }}{N}_{C{O}_{2}}^{g}+{\rm{\Delta }}{N}_{TC}\\  & = & (39.3{C}_{BC,f}^{l}{V}_{f}^{l}+{N}_{BC}^{r}-39.3{C}_{BC,0}^{l}{V}_{0}^{l})\\  &  & +\,({C}_{C{O}_{2},f}^{g}{V}_{f}^{g}+{N}_{C{O}_{2}}^{r}-{C}_{C{O}_{2},0}^{g}{V}_{0}^{g})+({C}_{TC,f}^{l}{V}_{f}^{l}+{N}_{TC}^{r}-{C}_{TC,0}^{l}{V}_{0}^{l})\,\end{array}$$

where ${V}_{0}^{l}$ and ${V}_{f}^{l}$ are the volumes of the initial and final liquid medium (in mL), respectively; ${C}_{BC,0}^{l}$ and ${C}_{BC,f}^{l}$ are initial and final biomass concentrations (in gDCW/L), respectively; ${N}_{BC}^{r}$ is the amount of biomass removed by sampling, which is the accumulation of all samples taken during the initial and final period: $\sum _{s}39.3{C}_{BC,s}^{l}{V}_{s}^{l}$. Other variables are defined in the similar fashion.

Mole balance suggests that ${N}_{C,Produced}={N}_{C,Consumed}$ between any two sampling points. Therefore, the percentage of consumed carbon that is accounted for by produced carbon in various products, as shown in Eqn. (7), can be used to evaluate the accuracy of various gas and liquid component concentration measurements in this work. Closer to 100% C accounted would indicate accurate measurements of various gas and liquid compoents.

7 $$ \% C\,\,Accounted=\frac{{N}_{C,Produced}}{{N}_{C,Consumed}}\times 100 \% $$

Detailed procedure and an example carbon balance calculation for biotic batch experiments can be found in Supplementary Information S3.

The total carbon balance for continuous cultures is similar to the total carbon balance for batch cultures, except that all terms of amounts are replaced by terms of rates. For consumed carbon,

8 $$\begin{array}{rcl}{F}_{C,Consumed} & = & {F}_{C{H}_{4},{\rm{in}}}-{F}_{C{H}_{4},{\rm{out}}}={C}_{C{H}_{4},{\rm{in}}}^{g}{Q}_{Total,\,\text{in}}^{g}-{C}_{C{H}_{4},{\rm{out}}}^{g}{Q}_{Total,\,{\rm{out}}}^{g}\\  & = & {y}_{C{H}_{4},{\rm{in}}}{F}_{Total,\,{\rm{in}}}^{g}-{y}_{C{H}_{4},{\rm{out}}}{F}_{Total,{\rm{out}}}^{g}\end{array}$$

where the feeding molar flow rate of CH_4_ (*i*.*e*., ${F}_{C{H}_{4},{\rm{in}}}$) is determined by mass flow controllers. The molar flow rate of CH_4_ in the off-gas (*i*.*e*., ${F}_{C{H}_{4},{\rm{out}}}$) is determined by CH_4_ concentration or mole fraction, which is measured by GC, and total volumetric or molar flow rate of the off-gas, which is determined by the proposed He tracer protocol as discussed previously.

For produced carbon,

9 $$\begin{array}{rcl}{F}_{C,Produced} & = & {F}_{BC}+{F}_{C{O}_{2}}^{g}+{F}_{TIC}+{F}_{OC}={F}_{BC}+{F}_{C{O}_{2}}^{g}+{F}_{TC}\\  & = & 39.3({C}_{BC,out}^{l}-{C}_{BC,in}^{l}){Q}^{l}+({C}_{C{O}_{2},out}^{g}{Q}_{Total,out}^{g}-{C}_{C{O}_{2},in}^{g}{Q}_{Total,in}^{g})\\  &  & +\,({C}_{TC,out}^{l}-{C}_{TC,in}^{l}){Q}^{l}\end{array}$$

where *F*’s are molar flow rates of different components; ${Q}_{Total,in}^{g}$ and ${Q}_{Total,out}^{g}$ are feed gas and off-gas volumetric flow rates; ${Q}^{l}$ is the liquid medium volumetric flow rate, which is assumed constant as pressure does not affect it appreciably. It is worth noting that when calculating produced carbon for continuous experiments, we do not need to consider the amount removed by gas or liquid sampling as the case for batch experiments. This is because the total liquid volume (therefore the headspace as well) is controlled at constant by adjusting the liquid effluent flow rate. In other words, the average effluent flow rate is lower than the average feed rate theoretically. However, because the difference is tiny considering only sampled in small amount every several hours, it is negligible.

Based on Eqs (8) and (9), $\% C\,Accounted$ defined below is used to evaluate the accuracy of various gas and liquid component concentration measurements.

10 $$ \% C\,Accounted=\frac{{F}_{C,Produced}}{{F}_{C,Consumed}}\times 100 \% $$

Detailed procedure and an example carbon balance calculation for biotic continuous experiments can be found in Supplementary Information S4.

## Results and Discussions

### Abiotic batch experiments comparing existing and proposed protocols

To examine the accuracy of the measured gas composition through different protocols for the abiotic systems, we compare the total amount of each gas component at the final state with that at the initial state. Following the computation procedure provided in the Methods section, *i*.*e*., Eqns (2) ∼ (4), the results are listed in Table 1 for Experiments 1 and 2. An example showing the detailed calculation procedure is provided in Supplementary Information S2.

**Table 1:** Comparison of the gas component measurement accuracy following the existing protocol (Experiment 1) and the proposed protocol (Experiment 2).

	Species	${{\boldsymbol{C}}}_{0}^{{\boldsymbol{g}}}$(mmol/L)	${{\boldsymbol{C}}}_{{\boldsymbol{f}}}^{{\boldsymbol{g}}}$(mmol/L)	*N*_*0*_(mmol)	*N*_*f*_(mmol)	*Err*%(*%*)
Exp. 1 (existing protocol)	CH_4_	4.10 ± 0.01	2.93 ± 0.01	0.43 ± 0.01	0.43 ± 0.01	−0.99 ± 0.07
	CO_2_	9.83 ± 0.05	7.93 ± 0.01	2.22 ± 0.01	2.18 ± 0.01	−2.21 ± 0.35
	O_2_	10.08 ± 0.01	9.11 ± 0.01	1.06 ± 0.01	1.32 ± 0.01	24.91 ± 0.05
Exp. 2 (proposed protocol)	CH_4_	3.02 ± 0.03	1.51 ± 0.16	0.29 ± 0.02	0.29 ± 0.02	−0.56 ± 2.36
	CO_2_	8.15 ± 0.03	4.65 ± 0.25	1.83 ± 0.01	1.79 ± 0.01	−2.22 ± 0.23
	O_2_	6.80 ± 0.02	3.54 ± 0.33	0.66 ± 0.03	0.68 ± 0.05	4.03 ± 2.00

The initial and final amount (*i*.*e*., ${N}_{0}$ and ${N}_{f}$) were calculated based on Eqs (2) and (3) respectively. Detailed calculation procedure can be found in Supplementary Information S2.

**Figure 2:**
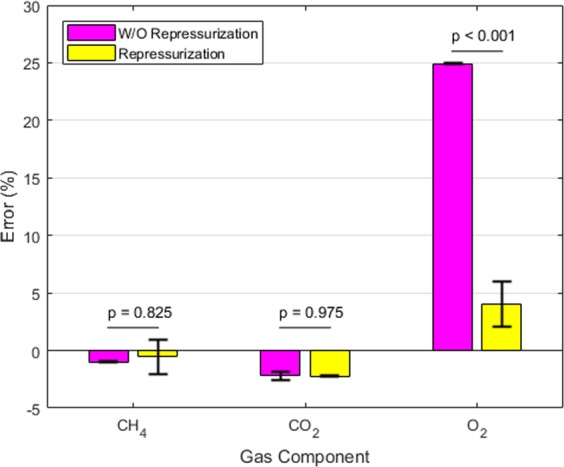
Comparison of gas component measurement errors based on the two protocols.

Table 1 shows that for the existing gas composition analysis protocol (*i*.*e*., Experiment 1), the measurement of O_2_ concentration contains significant error, as the final amount was significantly different from the initial amount; while the measurements of CH_4_ and CO_2_ were quite accurate with small errors. This was caused by the ambient air that entered the sampling syringe due to the vacuum pressure of the vials, as illustrated in Fig. 1. Because air contains no CH_4_ and less than 0.04% of CO_2_, it had no or minimum effect on the measurements of CH_4_ and CO_2_; however, since air contains 20.95% of O_2_, it caused large positive errors on the measurements of O_2_ concentration and amount. When following the proposed repressurization protocol for gas measurements (*i*.*e*., Experiment 2), Table 1 shows that for every gas component, there is no significant error. This demonstrates the effectiveness of the proposed protocol in ensuring accurate gas composition measurements for vacuum systems. The small positive error in O_2_ measurement may be attributed to the observed few air bubbles entering the vial under significant vacuum pressure while water was being removed through a syringe, plus the tiny amount of air already in the syringe needle before drawing gas samples. If desired, the latter can be avoided by flushing the syringe with an inert gas such as N_2_. It is worth noting that for CH_4_ and O_2_, the amount dissolved in liquid can be neglected due to their small solubility. However, it is not the case for CO_2_, as dissolved CO_2_ contributed more than half of the total amount as shown in the Supplementary Information S2. In addition, we performed student’s t-test to compare measurement errors of the three gas components under the two protocols. As shown in Fig. 2, the p-values of the t-test indicate that the measurement errors in CH_4_ and CO_2_ based on the two protocols are not statistically different, while the difference between the errors in O_2_ based on the two protocols are statistically significant at 99% confidence level.

### Biotic batch experiment with *M*. *buryatense* 5GB1

**Figure 3:**
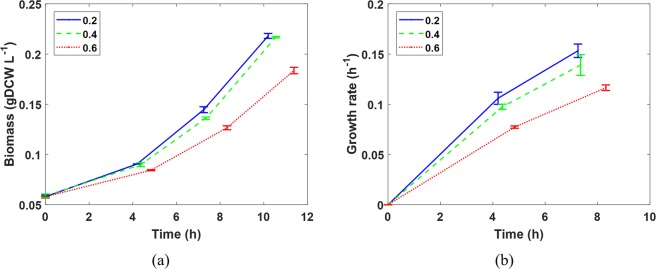
(**a**) Biomass accumulation and (**b**) specific growth rates of methanotrophs under different headspace gas compositions (denoted by the mole/volume fraction of O_2_ in the legend).

In this experiment we use batch mode bioconversion of methane with *M*. *buryatense* 5GB1 to examine the effectiveness of the proposed repressurization protocol. Three different feeding gas compositions were tested, with all of them containing the same amount of CH_4_ (20%), but different amount of O_2_ (20%, 40%, 60%) and N_2_ as the inert gas (60%, 40%, 20%). These feeding gas compositions correspond to three different O_2_:CH_4_ ratios of 1:1, 2:1 and 3:1. Figure 3(a) shows the biomass accumulation and Fig. 3(b) shows the specific cell growth rates within the first 12 hours. These plots clear show that higher oxygen content resulted in slower cell growth due to oxygen inhibition, which is in agreement with literature^13–15^.

Table 2 lists the total carbon balance results at the end of the batch experiment under different feeding gas conditions. It showed that close to 100% of the consumed carbon was accounted for all three conditions. The excellent total carbon balance results confirm that the proposed repressurization protocol is highly effective in ensuring accurate gas component concentration measurements via GC. The detailed carbon balance results, which include carbon contained in different products, are given in Supplementary Information S3. From S3, it is evident that pH has a significant impact on the distribution of CO_2_ between gas and liquid phase. For alkaline solutions, most of CO_2_ is dissolved in the liquid phase $(\frac{{\rm{\Delta }}{N}_{TIC}}{{\rm{\Delta }}{N}_{C{O}_{2}}^{g}+{\rm{\Delta }}{N}_{TIC}} \% =\,74 \%  \sim 85 \% )$; and the higher pH, the higher portion of dissolved CO_2_ in liquid phase. This general trend further confirms the validity and reliability of the repressurization protocol for measuring gas phase CO_2_ using GC as well as tracking liquid CO_2_ using TIC.

**Table 2:** Carbon balance achieved with repressurization protocol for batch biotic system.

pO_2_	pH	${{\boldsymbol{N}}}_{{\boldsymbol{C}},{\boldsymbol{Consumed}}}$ (mmol)	${{\boldsymbol{N}}}_{{\boldsymbol{C}},{\boldsymbol{Produced}}}$ (mmol)	%C Accounted (%)
0.2	8.61 ± 0.06	1.18 ± 0.01	1.19 ± 0.01	100.70 ± 1.27
0.4	8.27 ± 0.01	1.53 ± 0.01	1.53 ± 0.01	99.65 ± 0.64
0.6	8.22 ± 0.03	1.53 ± 0.01	1.51 ± 0.02	98.05 ± 1.34

### Biotic continuous experiment with *M*. *buryatense* 5GB1

**Figure 4:**
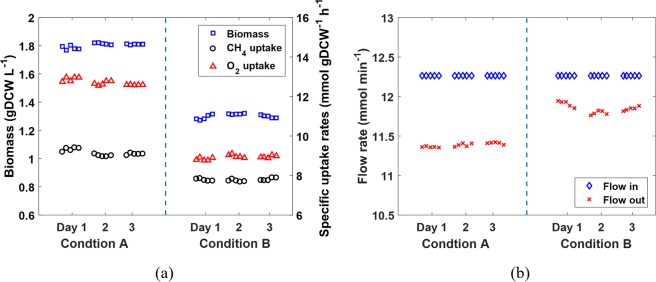
(**a**) Biomass and specific CH_4_, O_2_ uptake rates for the two chemostat conditions. (**b**) The feed and off-gas flow rates in comparison of one another for Conditions A and B.

In this section, we use *M*. *buryatense* 5GB1 cultured in a continuous stirred bioreactor to demonstrate the effectiveness of the proposed He tracer protocol. Specifically, the effects of carbon-limitation and oxygen-limitation on cell growth were evaluated, where carbon-limitation was denoted by condition A (24% O_2_,14% CH_4_ and 62% N_2_ with O_2_:CH_4_ ratio 1.71:1), and oxygen-limitation was denoted by condition B (12% O_2_, 14% CH_4_ and 74% N_2_ with O_2_:CH_4_ ratio 0.86:1). Figure 4(a) plots the steady-state measurements of biomass concentration, and CH_4_, O_2_ uptake rates under conditions A and B. Although we have developed an effective approach to accurately measure the volumetric mass transfer rate for O_2_^16^, *i*.*e*., ${k}_{La,{O}_{2}}$, in this work O_2_ uptake rate was determined through mole balance by measuring the feed and off-gas compositions. This is because ${k}_{La,{O}_{2}}$ is sensitive to biomass concentration and could vary significantly depending on experimental conditions^12^. Figure 4(b) plots the measured feed and off-gas flow rates through the He tracer protocol under culture conditions A and B. In this study, we assume a chemostat is achieved when the variation in biomass concentration is less than 10%. Since condition B contains the same amount of CH_4_ as in condition A but less amount of O_2_, lower biomass concentration is expected due to the same dilution rate, which is confirmed by Fig. 4(a). Figure 4(b) clearly shows that the off-gas flow rate could vary significantly from the feed gas flow rate. This significant difference demonstrates the importance of accurate measurement of the off-gas flow rate, which plays critical role in the total mass balance, *i*.*e*., Eq. (9).

Similar to the batch experiment, we use total carbon balance to validate the accuracy of the He tracer protocol. Through conducting a mole balance between adjacent measurements, substrate consumption rates, major product excretion rates were estimated and the total carbon balance is reported in Table 3. The carbon balance across different chemostats are consistently close to 100%, confirming the effectiveness of the proposed He tracer protocol in estimating off-gas flow rate.

**Table 3:** Consumption and production rates calculated based on the measurements following the helium tracer protocol result in consistent carbon balance under conditions A and B.

Condition	${{\boldsymbol{F}}}_{{\boldsymbol{C}},\text{Consumed}}$ (mmol C min^−1^)	${{\boldsymbol{F}}}_{{\boldsymbol{C}},{\boldsymbol{Produced}}}$ (mmol C min^−1^)	%C accounted (%)
A	24.72 ± 0.24	24.74 ± 1.37	100.11 ± 1.90
B	15.18 ± 0.13	15.71 ± 0.55	103.49 ± 1.73

### Difference between batch and continuous cultures

**Figure 5:**
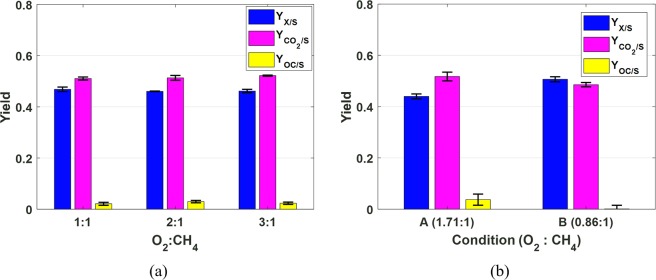
Comparison of different product yields obtained from the batch (**a**) and continuous (**b**) experiments.

With the reliable measurements of substrate uptake and product excretion rates, yield information can then be obtained with a high degree of confidence. Figure 5 compares the yields of different products obtained from batch and continuous experiments discussed in the previous subsections, where ${Y}_{X/S}$ denotes yield of biomass, ${Y}_{C{O}_{2}/S}$ yield of CO_2_, and ${Y}_{OC/S}$ yield of OC. For the biotic batch experiments, although different oxygen content results in different growth rate as shown in Fig. 3, it does not have much effect on how consumed carbon is distributed among different products as shown in Fig. 5(a). On the other hand, when cell growth rates are kept at chemostat in the continuous culture, different oxygen content has clear impact on how carbon is distributed among biomass, CO_2_ and OC as shown in Fig. 5(b). It is worth noting that without accurate measurements of gas component consumption and production rates, it is impossible to obtain reliable estimate of different yields. For the batch experiments, further analysis found that although O_2_:CH_4_ are provided in 1:1, 2:1 and 3:1 ratios, based on the reliable measurements of gas components, the actual O_2_:CH_4_ consumption ratios are 1.25:1, 1.31:1 and 1.31:1 respectively by averaging the replicates. The similar actual consumption ratios are consistent with the similar yield profile (*i*.*e*., ${Y}_{X/S}$, ${Y}_{C{O}_{2}/S}$ and ${Y}_{OC/S}$) across all experiments as shown in Fig. 5(a). These actual O_2_:CH_4_ consumption ratios are actually close to that of condition A in the continuous culture where the average O_2_:CH_4_ consumption ratio is 1.38:1. This is again in agreement with their similar yield profiles. On the other hand, condition B in the continuous culture has average O_2_:CH_4_ consumption ratio of 1.14:1, which resulted in significantly different yield profile as shown in Fig. 5(b). This finding also suggests that oxygen-limitation condition cannot be achieved in batch mode by simply controlling the O_2_:CH_4_ ratio in the headspace. No matter what ratio in the headspace, the cells will always go through aerobic growth till one substrate (O_2_ or CH_4_) in the headspace is completely consumed or reaches certain limit level. However, high O_2_ content does inhibit cell growth as shown in Fig. 3.

Statistical analyses were performed to further confirm the above observations. For the batch experiments, one-way analysis of variance (ANOVA) indicates that the differences among the yields of biomass under the three O_2_:CH_4_ ratios are statistically insignificant at 99% confidence level (p = 0.4908). Similarly, the differences among the yields of CO_2_ and OC under the three O_2_:CH_4_ ratios are statistically insignificant with p-values of 0.2727 and 0.3442, respectively. On the other hand, ANOVA of the continuous experimental data indicates that the differences of ${Y}_{X/S}$, ${Y}_{C{O}_{2}/S}$ and ${Y}_{OC/S}$ between conditions A and B are all statistically significant with p < 0.0001 for all three yields.

## Conclusion

In this work, we propose two experimental protocols and associated calculation procedures to enable accurate measurements of gas component consumption and production rates in bioconversion processes. One protocol is for depressurized batch systems, where an inert gas such as N_2_ or He is used to repressurize the system to 1 atm before sampling; the other is for continuous operations, where He is used as tracer or internal standard to obtain accurate measurement of the off-gas flow rate. Both abiotic systems and biotic systems with an aerobic methanotroph were used to demonstrate the effectiveness of the proposed protocols and calculation procedures in ensuring accurate consumption and production rate measurements of gas components. The accuracies of the gas component measurements based on both protocols were assessed through percentage error when the true values are known, or through total carbon balance when the true values are unknown. The proposed protocols and associated calculation procedures are easy-to-implement, do not require specialized equipment, and are generally applicable to various bioconversion processes where gas components are involved. We expect these protocols to have broad applications, and the accurately measured gas component consumption and production rates can help improve our quantitative understandings on many fundamental aspects of biological conversion processes.

## Supplementary information

Supplementary Information
