# Supplementary material for: Two Experimental Protocols for Accurate Measurement of Gas Component Uptake and Production Rates in Bioconversion Processes
Source: Sci Rep. 2019 Apr 11;9:5899. doi: 10.1038/s41598-019-42469-3 (PMC6459910; doi:10.1038/s41598-019-42469-3)
Supplement: Supplementary file 1 — Supplementary Information [file 41598_2019_42469_MOESM1_ESM.docx]

**Supplementary Information**

Two Experimental Protocols for Accurate Measurement of Gas Component Uptake and Production Rates in Bioconversion Processes

Kyle A. Stone, Q. Peter He^*^, Jin Wang^*^

Department of Chemical Engineering, Auburn University, Auburn, AL, USA 36849

***Correspondence**:

Jin Wang, Auburn University, 318 Ross Hall, Auburn AL 36849, USA Tel: 1-334-844-2020. Fax: 1-334-844-2063. Email: [wang@auburn.edu](mailto:wang@auburn.edu)

Q. Peter He, Auburn University, 344 Ross Hall, Auburn AL 36849, USA Tel: 1-334-844-7602. Fax: 1-334-844-2063. Email: [qhe@auburn.edu](mailto:qhe@auburn.edu)

# Henry solubility ($\boldsymbol{H}^{\boldsymbol{cc}}$) of the gases used in this study^8^

| Species | O_2_ | N_2_ | CH_4_ | CO2 (pH=7) | He |
| --- | --- | --- | --- | --- | --- |
| $\boldsymbol{H}_{\boldsymbol{i}}^{\boldsymbol{cc}}$ | 3.22×10^-2^ | 1.59×10^-2^ | 3.47×10^-2^ | 8.43×10^-1^ | 8.39×10^-3^ |

# Sample mole balance calculation for abiotic batch experiments:

Here we use the calculation of one abiotic experiment from Experiment 1 (*i.e.*, following the existing gas sampling protocol of without repressurization) to show the procedure. The calculations are repeated for all replicates to obtain the mean and standard deviations listed in Table 1.

- **Initial:** Experimental conditions: T=21^o^C; P=1atm; liquid (*i.e.*, water) phase volume: $V_{0}^{l}$=0.1589 L; gas phase volume: $V_{0}^{g}$=0.0911 L.

Gas phase is sampled and analyzed by GC to obtain the concentrations:

$C_{{CH}_{4},0}^{g}$ = 3.05 mmol/L; $C_{{CO}_{2},0}^{g}$ = 8.12 mmol/L; $C_{O_{2},0}^{g}$ = 6.78 mmol/L

These values are listed as $C_{0}^{g}$ in Table 1 for the three gas components.

Dimensionless Henry solubilities of various gases ($H_{i}^{cc}$, defined as the ratio between the liquid-phase concentration of a species and its gas-phase concentration) are listed below:

Table 1. Henry solubility ($H^{cc}$) of the gases used in this experiment^8^

| Species | O_2_ | CH_4_ | CO_2_ (pH=7) |
| --- | --- | --- | --- |
| $\boldsymbol{H}^{\boldsymbol{cc}}$ | 3.**22**×10^-2^ | 3.**47**×10^-2^ | 8.**43**×10^-1^ |

Based on Eqn. (2),

$N_{{CH}_{4},0}=C_{{CH}_{4},0}^{g}V_{0}^{g}+\left( c_{{CH}_{4},0}^{g}H_{{CH}_{4}}^{cc} \right)V_{0}^{l}$= (4.09)(0.10)+(4.09)(3.47×10^-2^)(0.15) = 0.430 (mmol)

Similarly,

$N_{{CO}_{2},0}=$(9.89)( 0.10)+(9.89)(8.43×10^-1^)( 0.15) = 2.24 (mmol)

$N_{O_{2},0}=$(10.08)( 0.10)+(10.08)(3.22×10^-2^)( 0.15) = 1.06 (mmol)

These values are listed as $N_{0}$ in Table 1 for the three gas components.

- **Final:** 40 mL (0.04 L) of water was removed with an air-tight syringe. The vial pressure becomes vacuum (specific pressure not measured) due to the removal of water. Afterwards, liquid (*i.e.*, water) phase volume: $V_{f}^{l}$=0.11 L; gas phase volume: $V_{f}^{g}$=0.14 L

Gas phase is then sampled and analyzed by GC. In the process, because of the vacuum pressure in the vial and syringe, the air leakage into the sampling syringe is unavoidable. The concentrations of various gases obtained from GC are:

$C_{{CH}_{4},f}^{g}$ = 2.92 mmol/L; $C_{{CO}_{2},f}^{g}$ = 7.93 mmol/L; $C_{O_{2},f}^{g}$ = 9.11 mmol/L

These values are listed as $C_{f}^{g}$ in Table 1 for the three gas components.

Based on Eqn. (3),

$$N_{{CH}_{4},f}=C_{{CH}_{4},f}^{g}V_{f}^{g}+\left( C_{{CH}_{4},f}^{g}H_{{CH}_{4}}^{cc} \right)V_{f}^{l}+\left( C_{{CH}_{4},0}^{g}H_{{CH}_{4}}^{cc} \right)V^{r}$$

$N_{{CH}_{4},f}=$(2.92)(0.14)+(2.92)(3.47×10^-2^)(0.11)+(4.09) (3.47×10^-2^)(0.04)=0.426 (mmol)

Similarly,

$N_{{CO}_{2},f}=$(7.93)(0.14)+(7.93)( 8.43×10^-1^)(0.11)+(9.89) (8.43×10^-1^)(0.04)=2.18 (mmol)

$N_{O_{2},f}=$(9.11)(0.14)+( 9.11)( 3.22×10^-2^)(0.11)+( 10.08) (3.22×10^-2^)(0.04)=1.32 (mmol)

These values are listed as $N_{f}$ in Table 1 for the three gas components.

- **Error:**

For CH_4_: $Err\%=\frac{{N_{{CH}_{4},f}-N}_{{CH}_{4},0}}{N_{{CH}_{4},0}}\times100\%=\frac{0.426-0.430}{0.430}\times100\%=-0.93\%$

Similarly,

For CO_2_: $Err\%=\frac{2.18-2.24}{2.24}\times100\%=-2.68\%$

For O_2_: $Err\%=\frac{1.32-1.06}{1.06}\times100\%=24.53\%$

These values are listed as $Err (\%)$ in Table 1 for the three gas components.

The above calculations are repeated for all replicates and the means and standard deviations are reported in Table 1.

The calculation procedure for Experiment 2 (*i.e.*, following the proposed gas sampling protocol of repressurization with N_2_) is the same as that for Experiment 1. The small $Err\%$ for O_2_ in Experiment 2 is solely due to the fact that the proposed repressurization protocol prevented air leaking into the sampling syringe during gas sample transfer from the system to the GC.

# Sample carbon balance calculation for biotic batch experiments:

Here we use the carbon balance calculation of one biotic batch experiment to show the procedure. The calculations are repeated for all replicates to obtain the means and standard deviations listed in Table 2.

Experimental conditions: T=21^o^C; P=1atm;

Initial liquid medium volume: $V_{0}^{l}$=0.0522 L; gas phase volume: $V_{0}^{g}$=0.1908 L.

Initial gas phase analyzed by GC to obtain the concentrations:

$C_{{CH}_{4},0}^{g}$ = 8.142 mmol/L; $C_{O_{2},0}^{g}$ = 8.071 mmol/L;$C_{{CO}_{2},0}^{g}$ = 0 mmol/L;

Initial biomass concentration: $C_{BC,0}^{l}=0.0591$ gDCW/L

Initial TC and TIC concentration:$C_{TC,0}^{l}=C_{TIC,0}^{l}=560.91$ mgC/L$=46.70$ mmolC/L

Final liquid medium volume: $V_{f}^{l}$ = 0.0316 L; gas phase volume: $V_{f}^{g}$ = 0.2114 L.

Final gas phase analyzed by GC following the proposed repressurization protocol to obtain the concentrations:

$C_{{CH}_{4},f}^{g}$ = 1.801 mmol/L; $C_{O_{2},f}^{g}$ = 0.466 mmol/L;$C_{{CO}_{2},f}^{g}$ = 0.430 mmol/L;

There was no gas sampling during the experiment. Therefore, based on Eqn. (5), the total amount of consumed carbon is:

$$N_{C,Consumed}=-\Delta N_{{CH}_{4}}^{g}=C_{{CH}_{4},0}^{g}V_{0}^{g}-C_{{CH}_{4},f}^{g}V_{f}^{g}=(8.142)(0.1908)-(1.801)(0.2114)$$

$=1.173$mmolC

Liquid phase were sampled six times during experiment. The weight of each sample is determined by weighing the vial before and after sampling. The volume of each sample is then determined based on the measured medium density of 1.008 g/mL. Biomass dry weight is obtained by OD measurement and its relationship to biomass concentration. The total biomass sampled/removed is the summation of all six samples, which is $4.51\times{10}^{-3}$ gDCW. Therefore, the total carbon in the sampled biomass is:

$N_{BC}^{r}=39.3(4.51\times{10}^{-3})=0.177$ mmolC

TC and TIC were measured for each sample and TOC was obtained as TC – TIC. The total TC removed is the summation of all six samples. For this experiment, $N_{TC}^{r}=1.079$ mmolC, $N_{TIC}^{r}=1.072$ mmolC

Final biomass concentration $C_{BC,f}^{l}=0.4091$ gDCW/L

Final TC concentration $C_{TC,f}^{l}=719.72$ mgC/L$=59.93$ mmolC/L; TIC concentration $C_{TC,f}^{l}=713.42$ mgC/L$=59.40$ mmolC/L.

Based on Eqn. (6), the total amount of carbon produced is:

$$N_{C,Produced}={\Delta N}_{BC}+{\Delta N}_{{CO}_{2}}^{g}+\Delta N_{TIC}+\Delta N_{TOC}={\Delta N}_{BC}+{\Delta N}_{{CO}_{2}}^{g}+\Delta N_{TC}$$

$${\Delta N}_{BC}=39.3C_{BC,f}^{l}V_{f}^{l}+N_{BC}^{r}-{39.3C}_{BC,0}^{l}V_{0}^{l}$$

$=(39.3)(0.4091)(0.0316)+0.177-(39.3)(0.0591)(0.0522)=0.564$ mmolC

${\Delta N}_{{CO}_{2}}^{g}=C_{{CO}_{2},f}^{g}V_{f}^{g}-C_{{CO}_{2},0}^{g}V_{0}^{g}=(0.430)(0.2144)=0.092$ mmolC

$${\Delta N}_{TC}=C_{TC,f}^{l}V_{f}^{l}+N_{TC}^{r}-C_{TC,0}^{l}V_{0}^{l}=\left( 59.93 \right)\left( 0.0316 \right)+1.079-\left( 46.70 \right)\left( 0.0522 \right)$$

$=0.535$ mmolC

Therefore, $N_{C,Produced}=0.564+0.092+0.535=$1.191 mmolC, and based on Eqn. (7)

$$\%C \text{Acco}\text{unted}=\frac{1.191}{1.173}\times100\%=101.53\%$$

The above calculations are repeated for all replicates to obtain the means and standard deviations listed in Table 2.

In addition, the amount of CO_2_ dissolved in the liquid phase can be estimated as

$${\Delta N}_{TIC}=C_{TIC,f}^{l}V_{f}^{l}+N_{TC}^{r}-C_{TIC,0}^{l}V_{0}^{l}=\left( 59.40 \right)\left( 0.0316 \right)+1.072-\left( 46.70 \right)\left( 0.0522 \right)$$

$=0.511$ mmolC

It can be seen that for alkaline solutions, most of CO_2_ is dissolved in the liquid phase $\frac{{\Delta N}_{TIC}}{{\Delta N}_{{CO}_{2}}^{g}+{\Delta N}_{TIC}}\%=$84.7% in this case.

# Sample carbon balance calculation for biotic continuous experiments:

Here we use the carbon balance calculation of one biotic continuous experiment at one sample time instant to show the procedure. The calculations are repeated for all sample time instants to obtain the means and standard deviations listed in Table 3.

Experimental conditions: T=22.1^o^C; P=1atm;

Entering gas component concentrations were controlled by mass controllers:

$Q_{{CH}_{4},in}^{g}$ = 42 smL/min; $Q_{O_{2},in}^{g}$ = 70 smL/min;$Q_{N_{2},in}^{g}$ = 158 mmol/L;$Q_{He,in}^{g}$ = 30 smL/min;

Therefore, $Q_{Total,in}^{g}$ = 300 smL/min and $F_{Total,in}^{g}$ = 12.263 mmol/min. In addition, the mole fraction of each gas component is obtained:

$y_{{CH}_{4},in}=\frac{42}{300}=0.140$; Similarly, $y_{O_{2},in}=0.233$; $y_{N_{2},in}=0.527$; and $y_{He,in}=0.100$

The mole flow rate of each gas component:

$F_{{CH}_{4},in}^{g}=y_{{CH}_{4},in}F_{Total,in}^{g}=$1.717 mmol/min

Similarly, $F_{O_{2},in}^{g}=2.861$ mmol/min; $F_{He,in}^{g}=1.226$ mmol/min

Off-gas analyzed by GC to obtain the concentrations of the off-gas components:

$C_{{CH}_{4},out}^{g}$ = 4.687 mmol/L; $C_{O_{2},out}^{g}$ = 8.243 mmol/L;$C_{{CO}_{2},out}^{g}$ = 0.153 mmol/L;$C_{He,out}^{g}$ = 4.434 mmol/L

To estimate the total effluent molar flow rate based on the proposed He tracer protocol, we assume ideal gas law is valid due to the low pressure, then

$y_{{CH}_{4},\text{out}}=\frac{C_{{CH}_{4},out}^{g} RT}{P}=0.114$; Similarly $y_{O_{2},\text{out}}=0.200$; $y_{{CO}_{2},\text{out}}=0.00370$; $y_{He,\text{out}}=0.107$

Mole balance based on He:

$$y_{He,\text{in}}F_{Total,in}^{g}={y_{He,\text{out}}F}_{Total,out}^{g}$$

Therefore,

$F_{Total,out}^{g}=\frac{y_{He,\text{in}}}{y_{He,\text{out}}}F_{Total,in}^{g}=11.416$ mmol/min

We can also find

$Q_{Total,out}^{g}=\frac{y_{He,\text{in}}}{y_{He,\text{out}}}Q_{Total,in}^{g}=280.37$ smL/min

As can be seen, $F_{Total,out}^{g}$ is different from $F_{Total,in}^{g}$, or $Q_{Total,out}^{g}$ is different from $Q_{Total,in}^{g}$.

Based on the total mole flow rate determined by the He tracer protocol, mole flow rates of other components can be determined:

$F_{{CH}_{4},out}^{g}=y_{He,\text{out}}F_{Total,out}^{g}=1.296$ mmol/min

Therefore, based on Eqn. (8),

${F_{C,Consumed}=F_{{CH}_{4},in}^{g}-F}_{{CH}_{4},out}^{g}=0.421$ mmol/min = $0.421$ mmolC/min

At the sample time instant, OD_600_ was measured as 0.6525 after seven-fold dilution, its concentration calculated based on calibration curve is:

$C_{BC,\mathrm{out}}=1.820$ gDCW/L

The volumetric flow rate of the liquid phase, $Q^{l}=2.64$ mL/min

Therefore, mole production rate of biomass

$F_{BC}=39.3(C_{BC,\mathrm{out}}{-C_{BC,\mathrm{in}})Q}^{l}=\left( 39.3 \right)\left( 1.820 \right)\left( 2.64\times{10}^{-3} \right)=0.189$ mmolC/min

The entering medium TC was measured at 94.701 mgC/L or 7.885 mmolC/L. The TC in the effluent liquid was measured at 949.049 mgC/L or 79.022 mmolC/L. Therefore, mole production rate of total carbon in the liquid other than biomass

$F_{TC}=\left( C_{TC,\text{out}}-C_{TC,\text{in}} \right)Q^{l}=\left( 79.022-7.885 \right)\left( 2.64\times{10}^{-3} \right)=0.188$ mmolC/min

Mole production rate of CO_2_ in the gas phase

$$F_{{CO}_{2}}^{g}=C_{{CO}_{2},out}^{g}Q_{Total,out}^{g}-C_{{CO}_{2},in}^{g}Q_{Total,in}^{g}=y_{{CO}_{2},\text{out}}F_{Total,out}^{g}-y_{{CO}_{2},\text{in}}F_{Total,in}^{g}$$

$=\left( 3.70\times{10}^{-3} \right)\left( 11.416 \right)=0.0422$ mmolC/min

because there was no CO_2_ in the feeding gas.

Therefore, based on Eqn. (9),

$F_{C,\text{Produced}}=F_{BC}+F_{TC}+F_{{CO}_{2}}^{g}=0.189+0.188+0.0422=$0.419 mmolC/min

Based on Eqn. (10),

$$\%C \text{A}\text{ccounted}=\frac{0.419}{0.421}\times100\%=99.5\%$$

The above calculations are repeated for all sampling points to obtain the means and standard deviations listed in Table 3.

It is worth noting that as shown in S3, the majority of the produced CO_2_ is dissolved in the liquid medium and is accounted for as part of TC. We did not differentiate TIC from TOC in carbon balance. However, we did differentiate them when calculating their yields in the paper.
